# The impact of cancer on diabetes outcomes

**DOI:** 10.1186/s12902-019-0377-0

**Published:** 2019-06-11

**Authors:** Anne Beiter Arreskov, Maria Å. Olsen, Sandra Sinius Pouplier, Volkert Siersma, Christen L. Andersen, Søren Friis, Niels de Fine Olivarius

**Affiliations:** 1 The Research Unit for General Practice and Section of General Practice, Department of Public Health, University of Copenhagen, Øster Farimagsgade 5, P.O. Box 2099, DK-1014 Copenhagen K, Denmark; 2 Danish Cancer Society Research Center, Danish Cancer Society, Copenhagen, Denmark

**Keywords:** Type 2 diabetes, Diabetes care, Cancer, Diabetes complication, All-cause mortality, Primary care, Randomized controlled trial

## Abstract

**Background:**

Survival from many cancer types is steadily increasing, and as a result, a growing number of cancer patients will live with other chronic diseases, of which diabetes is one of the most prevalent. This study aims to describe the impact of cancer on health outcomes in patients with type 2 diabetes and to compare the effectiveness of a multifactorial intervention in diabetes patients with and without cancer.

**Methods:**

The randomized controlled trial Diabetes Care in General Practice (DCGP) included 1381 patients newly diagnosed with type 2 diabetes. Patients were randomized to either six years of structured personal diabetes care or routine care. In a post hoc analysis, we followed patients for 19 years in Danish national registries for the occurrence of diabetes-related outcomes. We used Cox regression models to estimate hazard ratios for outcomes.

**Results:**

At diagnosis 48 patients had cancer, and 243 patients were diagnosed with cancer during follow up. Patients with diabetes and cancer had excess all-cause mortality (HR 3.33; 95%CI 2.72–4.06), as well as an increased incidence of myocardial infarction (HR 1.76; 95%CI 1.29–2.39) and any diabetes-related outcome (HR 1.36; 95%CI 1.07–1.71). The intervention reduced the risk of both these endpoints in patients without cancer. Furthermore, there was no statistically significant difference in the effectiveness of the intervention among patients with and without cancer.

**Conclusions:**

Diabetes patients with cancer had an increased risk of myocardial infarction and any diabetes-related outcome. The observed positive effect of structured personal diabetes care on clinical outcomes did not differ between patients with and without cancer. Attention to and prevention of diabetes complications in patients with both type 2 diabetes and cancer is warranted.

**Trial registration:**

ClinicalTrials.gov NCT01074762 (February 24, 2010).

**Electronic supplementary material:**

The online version of this article (10.1186/s12902-019-0377-0) contains supplementary material, which is available to authorized users.

## Background

Survival from many cancer types is steadily increasing [1]. As a result, a growing number of patients with cancer will live with other chronic diseases, of which diabetes is one of the most prevalent [2]. Besides giving rise to diabetes-related complications and death [3], type 2 diabetes in itself represents a risk factor for developing several types of cancer, e.g., pancreatic, liver, colorectal, breast, urinary tract, and female reproductive cancers [4]. Therefore, the number of cancer patients with diabetes will increase disproportionately [5].

Treating patients with both cancer and diabetes to diminish symptoms and improve quality of life is important. However, the management of diabetes in cancer patients is often complicated [6, 7]. For instance, the diagnosis and treatment of cancer may distract both patients, and health care providers form the appropriate management of diabetes. E.g., older studies show that regular chronic care of, e.g., DM. COPD and CVD is less common in patients who have completed primary cancer treatment compared to patients with no cancer [8–11], implying that for cancer patients with diabetes, both mortality and incidence of complications are increased compared to cancer patients without diabetes [12, 13].

Increased focus on improving the diabetes management of patients with both diabetes and cancer could lower the incidence of diabetes-related complications and mortality among these patients. However, while many studies describe how diabetes affects the incidence and mortality of cancer [14, 15], studies on the impact of cancer on diabetes-related outcomes are rare [8, 9].

Therefore, we studied the influence of a cancer diagnosis on the outcomes of diabetes management in the population-based cohort from the Diabetes Care in General Practice (DCGP) trial [16]. In this trial patients with newly diagnosed type 2 diabetes were randomized to receive either structured personal diabetes care or routine care. The intervention period lasted 6 years and patients were managed by their general practitioners (GPs). Results from this study showed that the intervention had a long-term effect on myocardial infarction (MI) and on the aggregate endpoint any diabetes-related outcomes.

The present analysis is a 19-year follow-up and observational post hoc analysis. The aim was to document the impact of a cancer diagnosis on various outcomes in patients with type 2 diabetes, who took part in the DCGP trial. In addition, we compared the effectiveness of structured personal diabetes care in patients with and without cancer. Outcomes were all-cause mortality, diabetes-related mortality, as well as cardiovascular and microvascular complications.

## Methods

A description of the study design has also been reported earlier [16–18]. The DCGP study was a cluster-randomized, open, controlled trial of the effect of intensified diabetes management (ClinicalTrials.gov NCT01074762), and the study adheres to CONSORT guidelines. A total number of 474 GPs volunteered to participate. Practices were randomly allocated to provide either routine care or structured personal care to their patients, who were newly diagnosed with type 2 diabetes.

### Patients

The GPs included all of their patients who were 40 years or older and diagnosed with diabetes during the inclusion period. At a major laboratory, the diabetes diagnosis was confirmed by a single fasting whole blood/plasma glucose concentration (≥7.0/8.0 mmol/l). The protocol-based exclusion criteria were: unwillingness to participate, severe psychiatric disease or life-threatening somatic disease (Additional file 1: Figure S1). As previously reported, the randomization was balanced [16, 17]. In total 1369 (99.1%) of 1381 patients in the final study population were of Western European descent. Approximately 97.5% of the patients were classified as type 2 diabetes patients, which was based on the onset of insulin treatment. The Frederiksberg and Copenhagen Research Ethics Committee approved the study.

### Intervention

The follow-up of patients in the intervention group consisted of examinations every three months and annual screening for diabetes complications. These examinations were facilitated by a questionnaire forwarded to the GP one month before the next expected consultation. The GP together with the patient was asked to define optimal goals for controlling important risk factors within three categories: ‘good,’ ‘acceptable,’ and ‘poor’ control. The emphasis was on glycemic control. At each quarterly consultation, GPs should compare the patient’s achievements with the goal and consider changing either goal or treatment accordingly. In overweight patients, the GP was prompted to agree with the patient on a small, realistic weight reduction, and this agreement should be recorded and followed up. However, participants were not required to target a particular body weight [16].

Through folders and leaflets for both physicians and patients, annual descriptive feedback reports on individual patients as well as six annual half-day seminars, GPs were introduced to possible solutions to therapeutic problems in the intervention group.

Generally, the importance of diet was stressed, and if possible, the GPs were recommended to postpone the start of glucose-lowering drugs until at least three months after diabetes diagnosis, to observe the effect of any weight loss. Further, the GPs were also prompted to recommend increased physical exercise and simple dietary rules [16, 17]. In cases of persistent hyperglycemia, metformin was recommended for patients who were overweight by clinical judgment. Glipizide or glibenclamide was suggested for patients of normal weight, and tolbutamide was recommended in patients older than 70 years. If the goal for blood glucose was not achieved and before starting insulin, a combination of metformin and a sulfonylurea was suggested as the last step. The preferred treatments for patients with hypertension were ACE-inhibitors or β-blockers; however, for patients with heart failure furosemide was preferred, and for patients older than 70 years thiazides were recommended. In cases of diet-resistant dyslipidemia, lipid-lowering drugs were recommended. To individualize the treatment, the GPs were allowed to deviate from the recommendations.

During the intervention phase, the GPs in the routine care group were free to choose any treatment and also to revise it [16]. The intervention was terminated on 26 September 1995, and the six-year examination was initiated. The study coordinators did not contact routine care practices during the intervention period after recruitment was completed and no attempt was made to maintain patients in randomized groups or to influence their therapy in the post-intervention period.

### Clinical and registry-based follow-up

A description of all variables and definitions has previously been published [16–19]. After median (IQR) 5.57 (4.96–6.16) years in the structured personal care group and after 5.85 (5.30–6.45) years in the routine care group, a clinical follow-up examination was completed for 970 (93.4%) of 1039 surviving patients (Additional file 1: Figure S1).

Using the unique identification number assigned to all Danish residents in the Danish Civil Registration System, emigration and vital status of all patients were ascertained. This enabled unambiguous linkage between the study population and the Danish national registries [20]. On 31 December 2008, all surviving patients were censored. The Danish Register of Causes of Death supplied information about possible and underlying contributory causes of death [21]. In four patients, the cause of death was not recorded. Information on cancer diagnoses was obtained from The Danish Cancer Registry [22], however non-melanoma skin cancer and some ill-defined cancers were not included in our cancer diagnosis (Additional file 1: Table S1). The Danish National Patient Register gave information on contacts with hospitals in Denmark, e.g., surgical procedures performed and discharge diagnoses [23]. These registries provided information on the predefined outcomes: all-cause mortality, diabetes-related deaths, any diabetes-related endpoint, stroke, myocardial infarction, microvascular disease, and peripheral vascular disease [16] (Additional file 1: Table S2).

### Statistical analysis

A description of the study design has also been reported earlier [16–18]. The incidence rates of the outcomes defined above were compared between structured care and routine care with hazard ratios (HRs) from Cox regression models on time from diagnosis to the first occurrence of the outcome; death and end of follow-up were censoring events. In these models, the accrual of a cancer diagnosis was modeled as a time-varying covariate. These comparisons were performed for patients with and without a cancer diagnosis separately to be able to assess whether there is a differential effect. Concurrently, in an auxiliary Cox regression analysis, the incidence rates for cancer were compared between structured care and routine care; these incidences are visualized in Kaplan-Meier curves. If a patient had an occurrence of an outcome before the diabetes diagnosis, this patient was excluded from the analyses about that outcome. In all assessments, we used a robust sandwich estimator to determine 95% CIs and *P* values to adjust for the clustering of patients within practices [24]. We further adjusted the comparisons for the following variables, assessed at diagnosis: sex, age, body mass index, hypertension, diagnostic fasting plasma glucose, total cholesterol, living alone, basic school education, sedentary physical activity, and current smoking. Incidence rates were calculated as the number of patients experiencing the corresponding outcome divided by the total person-time at risk. Patients with missing values in one or more variables were omitted from analyses where these variables were included.

Comparisons between structured care and routine care were done according to the intention-to-treat principle. Analyses were done using SAS (version 9.4). The level of statistical significance was 5%.

## Results

**Fig. 1:**
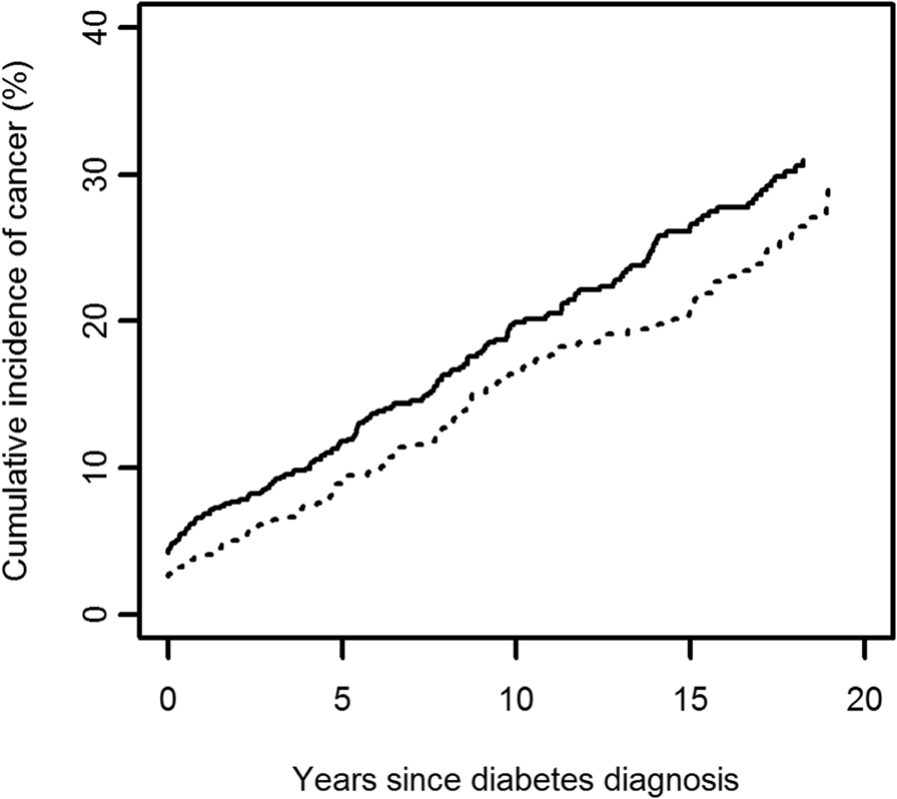
The cumulative incidence of cancer during 20 years after diabetes diagnosis according to randomization arm. Kaplan–Meier curves. Solid line: structured personal care; dotted line: routine care

At diabetes diagnosis, 48 of 1381 patients had a cancer diagnosis and the distribution of several characteristics, e.g., gender, fasting triglycerides, and current smoking seem to be unbalanced in relation to randomization arm in this small group (Tables 1 and 2). At the end of the intervention period, on average six years after the diagnosis of type 2 diabetes, 67 of 970 surviving and re-examined patients had cancer. At this point, levels of many of the clinical characteristics in these 67 patients (e.g., HbA_1c_ and blood pressures) was similar to those of the whole study population [16, 17] (Tables 1 and 2). This indicates that the effect of the intervention on intermediate outcomes was similar for patients with and without concomitant cancer. During the entire follow-up period of 19 years, including 13 years of post-intervention follow up, 243 patients were diagnosed with cancer (Additional file 1: Table S1). The occurrence of cancer diagnoses in the two arms of the trial over the study period is shown in Fig. 1. The rate of new cases of cancer does not differ between the two arms (log-rank test *P* = 0.28). Also, in a Cox regression model comparing the cancer incidences in the two arms, adjusted for the same variables as in Table 3, there is no evidence for a difference: HR for structured personal care (95% CI) is 1.10 (0.84–1.43), *P* = 0.50.

**Table 1:** Characteristics of patients with cancer at diabetes diagnosis and after 6 years of intervention

	Diabetes diagnosis (*n* = 48)			After 6 years of intervention (*n* = 67)		
	*n* (structured care/routine)	Structured personal care	Routine care	*n* (structured care/routine)	Structured personal care	Routine care
Sociodemographic						
Age (years)	32/16	70.3 (8.4)	70.8 (7.7)	37/23	74.9 (7.8)	72.9 (9.9)
Male gender	32/16	13 (40.6)	10 (62.5)	40/27	17 (42.5)	13 (48.2)
Live alone^a^	32/16	14 (43.8)	5 (31.3)	34/22	18 (52.9)	10 (45.5)
Basic School education^a^	32/14	30 (93.8)	13 (92.9)	39/25	32 (82.1)	19 (76.0)
Clinical						
Body mass index (kg/m^2^)	31/16	28.9 (4.4)	28.9 (3.9)	35/23	27.7 (5.0)	28.9 (4.7)
Hypertension	32/16	23 (71.9)	14 (87.5)	37/23	26 (70.3)	21 (91.3)
Systolic blood pressure (mmHg)	31/16	153.2 (25.5)	152.4 (16.0)	36/23	147.1 (20.7)	153.3 (15.3)
Diastolic blood pressure (mmHg)	31/16	83.7 (8.7)	85.8 (12.3)	36/23	78.2 (11.0)	84.1 (9.2)
Anti-diabetes treatment				37/23		
Diet only		–	–		11 (29.7)	9 (39.1)
Oral anti-diabetes treatment		–	–		23 (62.2)	9 (39.1)
Insulin		–	–		3 (8.1)	5 (21.7)
Biochemical						
Fasting plasma glucose^#^ (mmol/l)	32/16	13.1 (4.9)	12.2 (3.2)	30/17	9.3 (3.8)	11.1 (7.0)
Haemoglobin A1c (%)^b^	23/15	9.8 (2.5)	9.8 (1.7)	35/21	8.4 (1.5)	9.0 (2.0)
Total cholesterol (mmol/l)	30/16	6.3 (1.7)	6.1 (1.2)	35/21	6.2 (1.2)	6.1 (1.3)
Fasting triglycerides (mmol/l)	30/16	3.14 (6.4)	1.76 (0.7)	33/18	2.21 (1.3)	2.24 (1.2)
Serum creatinine (μmol/l)	30/16	98.5 (29.5)	104.3 (25.4)	35/21	98.4 (36.2)	99.0 (31.7)
Urinary albumin	28/16			33/19		
Normal		19 (67.9)	10 (62.5)		20 (60.6)	11 (57.9)
Microalbuminuria		9 (32.1)	6 (37.5)		11 (33.3)	8 (42.1)
Proteinuria		0 (0.0)	0 (0.0)		2 (6.1)	0 (0.0)
Behavioral^a^						
Sedentary physical activity	32/16	9 (28.1)	4 (25.0)	34/22	15 (44.1)	8 (36.4)
Current smoking	32/16	9 (28.1)	1 (6.3)	34/22	11 (32.4)	4 (18.2)
Patient attitudes^a^						
Self-rated health	32/16			34/22		
Excellent		3 (9.4)	1 (6.3)		5 (14.7)	3 (13.6)
Good		9 (28.1)	4 (25.0)		7 (20.6)	12 (54.6)
Fair		15 (46.9)	10 (62.5)		19 (55.9)	6 (27.3)
Poor/very poor		5 (15.6)	1 (6.3)		3 (8.8)	1 (4.6)
Process of care^a^						
No. of consultations last year		–	–	37/23	8.54 (4.7)	9.30 (6.2)
No. of diabetes-related consultations last year		–	–	37/23	4.19 (2.3)	4.26 (2.4)

Values are means (SD) or numbers (percentages of group). After 6 years of intervention, 27 of 48 patients with cancer at diagnosis were still alive and were re-examined together with 40 patients who were diagnosed with cancer during the intervention period. ^a^Data from questionnaires to patients (behavioral) or their general practitioners (process of care). ^b^The diagnostic value is limited to measurements from within 45 days of diabetes diagnosis. Reference range: 5.4–7.4%

**Table 2:** Characteristics of diabetes patients without cancer at diabetes diagnosis and after 6 years of intervention

	Diabetes diagnosis (*n* = 1333)			After 6 years of intervention (*n* = 1002)		
	*n* (structured care/routine)	Structured personal care	Routine care	*n* (structured care/routine)	Structured personal care	Routine care
Sociodemographic						
Age (years)	729/604	64.5 (11.6)	64.2 (11.4)	512/398	67.9 (11.0)	68.2 (10.9)
Male gender	729/604	391 (53.6)	319 (52.8)	556/446	17 (42.5)	13 (48.2)
Live alone^a^	711/590	222 (31.2)	193 (32.7)	472/382	163 (19.1)	132 (15.5)
Basic School education^a^	691/574	544 (78.7)	446 (77.9)	531/427	412 (43.0)	329 (34.3)
Clinical						
Body mass index (kg/m^2^)	722/603	29. (5.2)	29.5 (5.4)	502/387	29.0 (5.0)	28.8 (4.9)
Hypertension	729/604	545 (74.8)	444 (73.5)	512/398	371 (40.8)	291 (32.0)
Systolic blood pressure (mmHg)	724/603	149.2 (22.8)	147.3 (21.5)	510/392	146.8 (20.3)	151.4 (21.7)
Diastolic blood pressure (mmHg)	724/603	85.0 (10.9)	84.7 (10.8)	510/392	82.9 (9.4)	83.0 (10.7)
Anti-diabetes treatment				511/398		
Diet only		–	–		148 (29.0)	122 (30.6)
Oral anti-diabetes treatment		–	–		303 (59.3)	222 (55.8)
Insulin		–	–		60 (11.7)	54 (13.6)
Biochemical						
Fasting plasma glucose (mmol/l)	729/604	14.5 (5.4)	14.8 (5.9)	396/283	8.7 (3.4)	9.7 (3.7)
Hemoglobin A1c (%)^b^	601/497	10.3 (2.1)	10.3 (2.1)	504/393	8.7 (1.5)	9.2 (1.7)
Total cholesterol (mmol/l)	710/594	6.3 (1.5)	6.5 (1.7)	503/393	6.0 (1.4)	6.2 (1.2)
Fasting triglycerides (mmol/l)	706/594	2.6 (2.3)	2.9 (4.4)	470/338	2.2 (2.4)	2.3 (1.6)
Serum creatinine (μmol/l)	710/595	92.2 (19.1)	92.3 (21.9)	503/393	97.0 (51.8)	96.4 (29.5)
Urinary albumin	695/579			480/375		
Normal		400 (57.6)	331 (57.2)		298 (34.9)	218 (25.5)
Microalbuminuria		259 (37.3)	219 (37.8)		162 (19.0)	135 (15.8)
Proteinuria		36 (5.2)	29 (5.0)		20 (2.3)	22 (2.57)
Behavioral^a^						
Sedentary physical activity	709/588	201 (28.4)	158 (26.9)	466/379	128 (15.2)	120 (14.2)
Current smoking	710/588	255 (36.0)	207 (35.2)	470/377	151 (17.9)	112 (13.2)
Patient attitudes^a^						
Self-rated health	712/590			472/381		
Excellent		81 (11.4)	74 (12.5)		83 (9.73)	80 (9.4)
Good		243 (34.1)	192 (35.5)		206 (24.2)	141 (16.5)
Fair		321 (45.1)	259 (43.9)		158 (18.5)	144 (16.9)
Poor/very poor		67 (9.41)	65 (11.0)		25 (2.9)	16 (1.9)
Process of care^a^						
No. of consultations last year		–	–	512/397	8.2 (6.5)	7.2 (5.5)
No. of diabetes-related consultations last year		–	–	512/397	5.2 (3.4)	4.4 (3.6)

Values are means (SD) or numbers (percentages of group)

^a^Data from questionnaires to patients (behavioral) or their general practitioners (process of care)

^b^The diagnostic value is limited to measurements from within 45 days of diabetes diagnosis. Reference range: 5.4–7.4%

**Table 3:** Mortality and other outcomes during 19 years of follow up

Outcome	Incidence rate (events per 1000 patient years, 95% CI)		Hazard ratio (95% CI)^a^ for cancer versus no cancer^b^	*P* value^c^
	No cancer	Cancer		
All-cause mortality	48.3 (44.8–51.9)	226.7 (199.3–256.7)	3.33 (2.72–4.06)	< 0.0001
Diabetes-related deaths	34.2 (31.3–37.3)	87.7 (71.1–107.2)	1.81 (1.41–2.32)	<.0.0001
Any diabetes-related endpoint	64.3 (59.4–69.5)	418.7 (351.5–495.1)	1.36 (1.07–1.71)	0.011
Myocardial infarction	26.3 (23.6–29.2)	90.3 (71.2–113.1)	1.76 (1.29–2.39)	0.0003
Stroke	19.2 (16.9–21.7)	47.5 (34.3–64.3)	1.04 (0.68–1.57)	0.87
Peripheral vascular disease	4.16 (3.18–5.35)	7.54 (3.22–14.9)	0.92 (0.35–2.44)	0.86
Microvascular disease	10.4 (8.80–12.2)	33.4 (23.0–46.9)	1.44 (0.87–2.38)	0.16

^a^The hazard ratio (HR) is calculated in a Cox proportional hazard regression model where the first cancer diagnosis is a time-varying covariate. The corresponding 95% CI and *P* values are determined using a sandwich estimator for the variance to account for clustering of patients within practices

^b^Adjusted for age, sex and clustering, as well as for the following variables at diagnosis: live alone, basic school education, body mass index, hypertension, diagnostic fasting plasma glucose, total cholesterol, sedentary physical activity, and current smoking

^c^Tests the effect of cancer versus no cancer within patients with type 2 diabetes mellitus

The influence of prevalent or incident cancer

Independent of the randomization group, the incidence rate for each individual outcome was greater among cancer patients than among non-cancer patients during the 19 years of follow-up (Table 3). In multivariable-adjusted analyses, the excess all-cause mortality (HR 3.33) and diabetes-related mortality (HR 1.81) for cancer patients were confirmed. However, among the five remaining predefined outcomes, only the difference for MI (HR 1.76) and the aggregate outcome of any diabetes-related outcome (HR 1.36) were statistically significant (Table 3).

Among patients without cancer, the intervention reduced the risk of MI and any diabetes-related outcome, a result that is consistent with the findings in the main trial [16] (Table 4). We found no statistically significant difference in the effectiveness of the intervention between patients with and without cancer. Furthermore, there was no clear trend as to whether the intervention effect was larger or smaller among cancer patients.

**Table 4:** Mortality and other outcomes for patients with and without cancer in patients receiving structured or routine care during 19 years of follow up

	No. of patients with outcome during 19 years of follow up (*n* (%))		Incidence rate (events per 1000 patient years, 95% CI)		Hazard ratio^a^ (95% CI) for structured care versus routine care^b^	*P* value^c^	Inter-ac-tion *P* value^d^
	Structured personal care	Routine care	Structured personal care	Routine care			
All-cause mortality							
Cancer	147 (84.5)	101 (86.3)	220.1 (185.9–258.7)	237.0 (193.1–288.1)	0.97 (0.67–1.41)	0.89	0.65
No cancer	375 (63.9)	343 (68.2)	45.8 (41.3–50.7)	51.2 (45.9–56.9)	0.89 (0.76–1.03)	0.12	
Diabetes-related deaths							
Cancer	58 (33.5)	38 (32.5)	86.8 (65.9–112.3)	89.2 (63.1–122.5)	1.01 (0.63–1.63)	0.96	0.75
No cancer	269 (45.9)	240 (47.9)	32.8 (29.0–37.0)	35.8 (31.4–40.6)	0.93 (0.77–1.13)	0.46	
Any diabetes-related endpoint							
Cancer	79 (53.4)	58 (61.1)	339.8 (269.0–423.6)	612.5 (465.0–792.2)	0.75 (0.49–1.16)	0.19	0.81
No cancer	331 (70.6)	314 (75.3)	58.9 (52.8–65.7)	71.1 (63.5–79.4)	0.80 (0.68–0.93)	0.005	
Myocardial infarction							
Cancer	42 (25.2)	34 (31.2)	77.0 (55.5–104.1)	114.9 (79.5–160.7)	0.94 (0.53–1.69)	0.84	0.50
No cancer	177 (33.5)	176 (38.8)	23.7 (20.3–27.5)	29.5 (25.3–34.2)	0.76 (0.62–0.94)	0.011	
Stroke							
Cancer	23 (13.9)	19 (17.6)	43.4 (27.4–65.1)	53.9 (32.4–84.3)	0.65 (0.30–1.41)	0.27	0.38
No cancer	133 (23.7)	127 (26.3)	17.8 (14.9–21.1)	21.0 (17.5–25.0)	0.94 (0.74–1.11)	0.60	
Peripheral vascular disease							
Cancer	4 (2.3)	4 (3.5)	6.1 (1.6–15.8)	9.8 (2.6–25.4)	0.85 (0.49–1.48)	0.99	0.88
No cancer	31 (5.3)	30 (6.0)	3.8 (2.6–5.5)	4.6 (3.1–6.5)	0.99 (0.16–6.13)	0.57	
Microvascular disease							
Cancer	20 (11.6)	13 (11.1)	33.4 (20.4–51.7)	33.3 (17.7–56.2)	1.35 (0.51–3.57)	0.37	0.39
No cancer	78 (13.3)	70 (13.9)	9.9 (7.8–12.4)	11.0 (8.6–13.9)	0.85 (0.60–1.21)	0.55	

^a^The hazard ratio (HR) is calculated in a Cox proportional hazard regression model where the first cancer diagnosis is a time-varying covariate. The corresponding 95% CI and *P* values are determined using a sandwich estimator for the variance to account for clustering of patients within practices

^b^Adjusted for age, sex and clustering, as well as for the following variables at diagnosis: live alone, basic school education, body mass index, hypertension, diagnostic fasting plasma glucose, total cholesterol, sedentary physical activity, and current smoking

^c^Tests the effect of randomization within patient groups with and without cancer. ^d^Tests whether the effect of randomization is different between patient groups with and without cancer

The effectiveness of the intervention

## Discussion

To our knowledge, this is the first study to compare the effect of a diabetes intervention in diabetes patients with and without cancer. In this post hoc observational analysis with 19 years of follow up, we examined the effectiveness of structured personal diabetes care versus routine diabetes care in diabetes patients with and without cancer. Also, we evaluated the overall impact of cancer in these patients with newly diagnosed type 2 diabetes. Our results demonstrate that compared to diabetes patients without cancer, patients with both diabetes and cancer had significantly increased all-cause and diabetes-related mortality as well as increased incidence of any diabetes-related endpoint and MI. In the original report from the DCGP trial, as well as in the current study, the intervention was associated with a significantly reduced risk of MI and any diabetes-related endpoint in patients with type 2 diabetes [16]. However, diabetes patients with cancer benefitted neither more nor less from structured personal diabetes care compared to diabetes patients without cancer.

It is well documented that cancer patients with diabetes have higher mortality than cancer patients without diabetes [12, 25]. A Danish study found that for all cancers combined and diabetes duration of 2 years at cancer diagnosis, patients treated with insulin experienced the highest mortality rate ratios starting from 3.7 for men and 4.4 for women one year after the cancer diagnosis [12]. Our HR (3.3) for all-cause mortality is comparable with these results. Similarly, other studies have shown that patients with both cancer and diabetes have an increased risk of death from MI, compared to cancer patients without diabetes [26].

The interaction between diabetes and cancer is complex [27]. There is good evidence for biologic mechanisms (e.g., hyperinsulinemia, hyperglycemia, inflammatory pathways, oxidative stress, and changes in hormones) contributing to the increased mortality risk when the diseases occur simultaneously [28]. Further, certain shared risk factors may be involved, such as age, sex, body weight, diet, physical activity, alcohol, and smoking, as these risk factors are likely to influence diabetes treatment as well as diabetes-related outcomes. In addition, cardiovascular complications of cancer chemotherapy and radiation are common [29], as well as glucocorticoids used in cancer treatment may also interfere with glucose metabolism and cause diabetes, or worsen pre-existing diabetes. Further, some antidiabetic drugs have been under scrutiny because of their potential influences on cancer development in a population already at risk [30]. Biologic mechanisms, shared risk factors, as well as factors associated with treatment, may, therefore, contribute to the increased mortality in patients with diabetes and cancer.

Other factors, such as changes in priorities in the management of comorbidities in cancer patients, could also play a role in excess mortality. For instance, Sabatino et al. [10] found that many providers may miss opportunities to counsel cancer survivors about important behaviors regarding diet, exercise, and smoking, even though these behaviors may influence the prognosis of these patients. In like manner, patient priorities might also change, as patients focus on new, competing demands, while pre-existing conditions attain a lower priority [31]. E.g., studies have found that having a comorbid disease or receiving chemotherapy is likely to reduce patients’ prioritization and self-management of diabetes [32, 33]. Further, management of chronic diseases in patients with cancer is connected with lower testing rates [11] and lower adherence to medication [34, 35] resulting in difficulties meeting treatment goals for HbA_1c_, LDL-cholesterol and blood pressure targets [36].

In contrast, Chiao et al. compared diabetes outcomes one year before and after a colorectal cancer diagnosis in American veterans with diabetes [37]. They found that blood pressure and cholesterol levels remained unchanged, whereas HbA_1c_ levels showed a tendency to improve. In addition, other studies [38–41] showed that diabetes patients with cancer received diabetes care of generally similar quality compared to diabetes patients without cancer.

In the present study, the number of consultations among cancer patients in the two study arms showed no significant difference after six years of intervention.

### Strengths and limitations

The results from this post hoc analysis of a randomized controlled trial should be interpreted as observational, as the presence of cancer was not accounted for in the randomization procedure. In the period following a diabetes diagnosis, the enhanced medical examination could lead to increased detection of cancer, which has been reported in some studies [42] and rejected in another study [43]. However, in the present study, the intervention did not influence the detection of cancer (Fig. 1), as the rate of new cases of cancer did not differ between the two arms.

The distribution of types of cancers resulted in relatively small numbers of patients in different sub-classes of cancer (Additional file 1: Table S1), which prevented us from making subgroup analyses according to cancer type and stage. An observational study did not find any effect of tumor stage and site on diabetes quality indicators among cancer patients [38]. Nevertheless, it seems reasonable to assume that aggressive lung cancer or pancreatic cancer has a larger impact on the course of diabetes than limited skin cancers, which are not included in this study.

The group of patients who had cancer recorded before the diabetes diagnosis included only patients who had survived this cancer, and it did not include patients with terminal cancers as this was an explicit exclusion criterion. The group of patients who got a cancer diagnosis after the diabetes diagnosis did include more aggressive type of cancers. It is, therefore, a limitation of the present study that the effect of cancer was modeled without taking the timing of the cancer diagnosis into account. People who did not survive probably had more severe conditions and could have been treated less intensively as regards their diabetes and diabetic complications.

The cancer diagnoses were identified in the Danish Cancer Registry, which has been documented to have high completeness and accuracy [22, 44] (Additional file 1: Table S1).

## Conclusions

In this post hoc analysis of the DCGP trial, diabetes patients with cancer had significantly increased mortality, an increased incidence of myocardial infarction, as well as an increased incidence of the aggregate endpoint any diabetes-related outcome, compared to patients without cancer. The intervention reduced the risk of MI and any diabetes-related outcome in patients without cancer, and there were no statistically significant differences in the effectiveness of the intervention between patients with and without cancer. The observed high risk of cardiovascular disease among patients with both diabetes and cancer suggests that this late complication should receive extra attention in this group of high-risk patients.

## Additional file

Additional file 1: **Table S1.** Categorization of all patients from the Diabetes Care in General Practice (DCGP) study with ICD-10 diagnoses in the Danish Cancer Register (DCR). **Table S2.** Definition of clinical outcomes in the 19-year registry-based monitoring of the Diabetes Care in General Practice (DCGP) study. **Figure S1.** Patient flow through study. (DOCX 110 kb)

## Data Availability

The datasets used and analyzed during the current study are available from the corresponding author on reasonable request.

## Abbreviations

DCGP: Diabetes Care in General Practice
GP: General practitioner
HR: Hazard ratio
MI: Myocardial infarction

## References

[1] De Angelis R, Sant M, Coleman MP, Francisci S, Baili P, Pierannunzio D, Trama A, Visser O, Brenner H, Ardanaz E (2014). Cancer survival in Europe 1999-2007 by country and age: results of EUROCARE--5-a population-based study. Lancet Oncol.

[2] Pouwer F (2015). Impact of cancer on use of glucose-lowering drug treatment in individuals with diabetes: potential mechanisms. Diabetologia.

[3] de Fine Olivarius N, Andreasen AH (1997). Five-year all-cause mortality of 1323 newly diagnosed middle-aged and elderly diabetic patients. Data from the population-based study, diabetes care in general practice, Denmark. J Diabetes Complications.

[4] Onitilo AA, Engel JM, Glurich I, Stankowski RV, Williams GM, Doi SA (2012). Diabetes and cancer I: risk, survival, and implications for screening. Cancer Causes Control.

[5] Williams GR, Mackenzie A, Magnuson A, Olin R, Chapman A, Mohile S, Allore H, Somerfield MR, Targia V, Extermann M, et al. Comorbidity in older adults with cancer. J Geriatric Oncol. 2015. 10.1016/j.jgo.2015.12.002 PMC4917479 26725537

[6] Gallo M, Gentile L, Arvat E, Bertetto O, Clemente G (2016). Diabetology and oncology meet in a network model: union is strength. Acta Diabetol.

[7] Psarakis HM (2006). Clinical Chalenges in caring for patients with diabetes and Cancer. Diabetes Spectrum.

[8] Snyder CF, Frick KD, Herbert RJ, Blackford AL, Neville BA, Wolff AC, Carducci MA, Earle CC (2013). Quality of care for comorbid conditions during the transition to survivorship: differences between cancer survivors and noncancer controls. J Clin Oncol.

[9] Earle CC, Neville BA (2004). Under use of necessary care among cancer survivors. Cancer.

[10] Sabatino SA, Coates RJ, Uhler RJ, Pollack LA, Alley LG, Zauderer LJ (2007). Provider counseling about health behaviors among cancer survivors in the United States. J Clin Oncol.

[11] Yao N, Camacho FT, Chukmaitov AS, Fleming ST, Anderson RT (2015). Diabetes management before and after cancer diagnosis: missed opportunity. Ann Transl Med.

[12] Ranc K, Jorgensen ME, Friis S, Carstensen B (2014). Mortality after cancer among patients with diabetes mellitus: effect of diabetes duration and treatment. Diabetologia.

[13] Barone BB, Yeh HC, Snyder CF, Peairs KS, Stein KB, Derr RL, Wolff AC, Brancati FL (2008). Long-term all-cause mortality in cancer patients with preexisting diabetes mellitus: a systematic review and meta-analysis. Jama.

[14] Zhao XB, Ren GS (2016). Diabetes mellitus and prognosis in women with breast cancer: a systematic review and meta-analysis. Medicine.

[15] Sogaard M, Thomsen RW, Bossen KS, Sorensen HT, Norgaard M (2013). The impact of comorbidity on cancer survival: a review. Clin Epidemiol.

[16] Hansen LJ, Siersma V, Beck-Nielsen H, de Fine Olivarius N (2013). Structured personal care of type 2 diabetes: a 19 year follow-up of the study diabetes Care in General Practice (DCGP). Diabetologia.

[17] Pouplier S, Olsen MA, Willadsen TG, Sandholdt H, Siersma V, Andersen CL, Olivarius NF (2018). The development of multimorbidity during 16 years after diagnosis of type 2 diabetes. J Comorb.

[18] Larsen JR, Siersma VD, Davidsen AS, Waldorff FB, Reventlow S, de Fine Olivarius N (2016). The excess mortality of patients with diabetes and concurrent psychiatric illness is markedly reduced by structured personal diabetes care: a 19-year follow up of the randomized controlled study diabetes Care in General Practice (DCGP). Gen Hosp Psychiatry.

[19] Krag MO, Hasselbalch L, Siersma V, Nielsen AB, Reventlow S, Malterud K, de Fine Olivarius N (2016). The impact of gender on the long-term morbidity and mortality of patients with type 2 diabetes receiving structured personal care: a 13 year follow-up study. Diabetologia.

[20] Pedersen CB, Gotzsche H, Moller JO, Mortensen PB (2006). The Danish civil registration system. A cohort of eight million persons. Dan Med Bull.

[21] Helweg-Larsen K (2011). The Danish register of causes of death. Scand J Public Health.

[22] Gjerstorff ML (2011). The Danish cancer registry. Scand J Public Health.

[23] Lynge E, Sandegaard JL, Rebolj M (2011). The Danish National Patient Register. Scand J Public Health.

[24] Kalbfleisch JDPR (2002). The statistical analysis of failure time data.

[25] Renehan AG, Yeh HC, Johnson JA, Wild SH, Gale EA, Moller H (2012). Diabetes and cancer (2): evaluating the impact of diabetes on mortality in patients with cancer. Diabetologia.

[26] Liu X, Ji J, Sundquist K, Sundquist J, Hemminki K (2012). Mortality causes in cancer patients with type 2 diabetes mellitus. Eur J Cancer Prev.

[27] Klil-Drori AJ, Azoulay L, Pollak MN (2017). Cancer, obesity, diabetes, and antidiabetic drugs: is the fog clearing?. Nat Rev Clin Oncol.

[28] Koene RJ, Prizment AE, Blaes A, Konety SH (2016). Shared risk factors in cardiovascular disease and cancer. Circulation.

[29] Cameron AC, Touyz RM, Lang NN (2016). Vascular complications of Cancer chemotherapy. Can J Cardiol.

[30] Hua F, Yu JJ, Hu ZW (2016). Diabetes and cancer, common threads and missing links. Cancer Lett.

[31] Morris RL, Sanders C, Kennedy AP, Rogers A (2011). Shifting priorities in multimorbidity: a longitudinal qualitative study of patient's prioritization of multiple conditions. Chronic Illn.

[32] Kerr EA, Heisler M, Krein SL, Kabeto M, Langa KM, Weir D, Piette JD (2007). Beyond comorbidity counts: how do comorbidity type and severity influence diabetes patients’ treatment priorities and self-management?. J Gen Intern Med.

[33] Hershey DS, Tipton J, Given B, Davis E (2012). Perceived impact of cancer treatment on diabetes self-management. Diabetes Educ.

[34] Calip GS, Elmore JG, Boudreau DM (2017). Characteristics associated with nonadherence to medications for hypertension, diabetes, and dyslipidemia among breast cancer survivors. Breast Cancer Res Treat.

[35] Zanders MM, Haak HR, Van Herk-Sukel MP, van de Poll-Franse LV, Johnson JA (2015). Impact of cancer on adherence to glucose-lowering drug treatment in individuals with diabetes. Diabetologia.

[36] Shin JY, Shim HY, Jun JK (2014). Comparison of diabetes management status between cancer survivors and the general population: results from a Korean population-based survey. PLoS One.

[37] Chiao EY, Nambi PV, Naik AD (2010). The impact of diabetes process and outcome quality measures on overall survival in patients with co-morbid colorectal cancer. J Cancer Surviv.

[38] Keating LN, Zaslavsky MA, Herrinton JL, Selby VJ, Wolf ER, Ayanian ZJ (2007). Quality of diabetes care among cancer survivors with diabetes. Med Care.

[39] Policardo L, Barchielli A, Seghieri G, Francesconi P (2016). Does the hospitalization after a cancer diagnosis modify adherence to process indicators of diabetes care quality?. Acta Diabetol.

[40] Earle CC, Burstein HJ, Winer EP, Weeks JC (2003). Quality of non-breast cancer health maintenance among elderly breast cancer survivors. J Clin Oncol.

[41] Bayliss EA, Blatchford PJ, Newcomer SR, Steiner JF, Fairclough DL (2011). The effect of incident cancer, depression and pulmonary disease exacerbations on type 2 diabetes control. J Gen Intern Med.

[42] Johnson JA, Bowker SL, Richardson K, Marra CA (2011). Time-varying incidence of cancer after the onset of type 2 diabetes: evidence of potential detection bias. Diabetologia.

[43] Geier AS, Wellmann J, Wellmann I, Kajuter H, Heidinger O, Hempel G, Hense HW (2013). Cancer detection rates following enrolment in a disease management programme for type 2 diabetes. Diabetologia.

[44] Storm HH, Michelsen EV, Clemmensen IH, Pihl J (1997). The Danish Cancer registry--history, content, quality and use. Dan Med Bull.

