# Supplementary material for: The impact of cancer on diabetes outcomes
Source: BMC Endocr Disord. 2019 Jun 11;19:60. doi: 10.1186/s12902-019-0377-0 (PMC6560822; doi:10.1186/s12902-019-0377-0)

Online-Only Supplemental Material for the article: *The impact of cancer on diabetes outcomes 19 years after the diagnosis of type 2 diabetes*

Table S1

Categorization of all patients from the Diabetes Care in General Practice (DCGP) study with ICD-10 diagnoses in the Danish Cancer Register (DCR)

| **Category** | **ICD-10** | **Patients with a cancer diagnosis at diabetes diagnosis** | | **Patients diagnosed with cancer during 19 years of follow up after diabetes diagnosis** | |
| --- | --- | --- | --- | --- | --- |
|  |  | **Struc-tured personal care (n=761)** | **Routine care (n=620)** | **Struc-tured personal care (n=761)** | **Routine care (n=620)** |
| All cancer |  | 32 | 16 | 142 | 101 |
| Buccal cavity and pharynx | C00-C14; C462 | 2 | 0 | 1 | 2 |
| Digestive organs | C15-C26 | 3 | 3 | 46 | 31 |
| Respiratory system including thoracic organs | C30-39; C450 | 0 | 0 | 18 | 10 |
| Bones, joints and articular cartilage | C40-C41 | 0 | 0 | 0 | 0 |
| Skin | C43; C460 | 1 | 1 | 6 | 4 |
| Mesothelium and connective tissue | C451-C459; C461; C463; C467; C468; C469; C47-C49; B210 | 1 | 0 | 2 | 1 |
| Breast | C50 | 3 | 5 | 21 | 10 |
| Female genital organs | C51-C58 | 10 | 2 | 6 | 7 |
| Male genital organs | C60-C63 | 2 | 2 | 17 | 14 |
| Urinary tract | C64-C68; D090-D091; D301-D309; D411-D419 | 8 | 3 | 12 | 17 |
| Eye and central nervous system | C69-C72; C751-C753; D32-D33; D352-D354; D42-D43; D443-D445 | 0 | 0 | 1 | 2 |
| Endocrine glands | C73-C74; C750; C754-C759 | 0 | 0 | 0 | 0 |
| Malignant neoplasms, stated or presumed to be primary, of lymphoid, haematopoietic and related tissue ^a^ | C81-C96  D45-D46; D470-D471; D473-D475 | 2 | 1 | 9 | 4 |
| Malignant neoplasms of ill-defined, secondary and unspecified sites ^b^ | C76-C80 | 0 | 0 | 12 | 7 |
| No cancer | - | - | - | 587 | 503 |

^a^ For Hodgkin and non-Hodgkin lymphomas including ICD for Oncology (ICD-O) morphological codes within the range: 9590/3/ - 9597/3/; 9650/3/ - 9667/3/; 9670/3/ - 9729/3/ as well as: 97313, 97323, 97333 and 97343 in accordance to the recommendation of the Danish Cancer Register.

^b^ Metastases are only included if there is no registration of any primary neoplasm.Table S2*:* Definition of clinical outcomes in the 19-year registry-based monitoring of the Diabetes Care in General Practice (DCGP) study

|  | **Codes used to classify**  **cause of death or morbidity^*^** | | **Codes used to classify**  **surgical procedures^†^** | |
| --- | --- | --- | --- | --- |
|  | ICD-8 codes  (≤1993) | ICD-10 codes  (≥1994) | The Danish Classification of Surgical Procedures  (≤1995) | The Nordic Classification of Surgical Procedures  (≥1996) |
| ***Aggregate endpoints. Time to the first occurrence of any event in that category:*** |  | | | |
| **Diabetes-related death, i.e. death from:** |  | | | |
| Fatal myocardial infarction | 410-414 or 427.0 or 427.1 or 427.9 or 428 | I20-I25 or I50 |  |  |
| Fatal stroke | 430-438 | I60-I69 or G45 |  |  |
| Renal disease | 580-593 | N00-N06 or N08 or N10-N13 or N17-N20 or N25-N29 or E10.2 or E11.2 or E12.2 or E13.2 or E14.2 |  |  |
| Hyperglycaemia |  | E10.0 or E10.1 or E11.0 or E11.1 or E12.0 or E12.1 or E13.0 or E13.1 or E14.0 or E14.1 |  |  |
| Hypoglycaemia | 962.3 | E16.0 or E16.1 or E16.2 |  |  |
| Sudden death | 795 or 796.2 or 796. or 796.9 | R96-R99 |  |  |
| Peripheral vascular disease | 440.2 | I70.2 or E10.5 or E11.5 or E12.5 or E13.5 or E14.5 |  |  |
| **(Death from any cause)** |  | | | |
| **Vascular (cardiac) =**  **“Myocardial infarction”** |  | | | |
| Fatal myocardial infarction | As above | | | |
| Nonfatal myocardial infarction | 410 | I21 |  |  |
| Sudden death | As above | | | |
| **Vascular (stroke) =**  **“Stroke”** |  | | | |
| Fatal stroke | As above | | | |
| Nonfatal stroke | 430-434 or 436 | I60-I64 |  |  |
| **Peripheral vascular disease** |  | | | |
| Amputation |  |  | 8103 or 8104 or 8105 or 81080 or 81081 | NEQ or  NFQ or  NGQ or  NHQ |
| Death from peripheral vascular disease | As above | | | |
| **Microvascular disease** |  | | | |
| Nonfatal renal failure | 792 | N18-N19 | 94340 or 94300 | TJA 30 |
| Fatal renal disease | As above | | | |
| Vitreous haemorrhage | 377.00 | H43.1 or H45.0 | 16540 | CKD65 |
| Retinal photocoagulation |  |  | 16070 | CKC10 or CKC15 |
| **Any diabetes-related end point** |  | | | |
| Sudden death | As above | | | |
| Death from hyperglycaemia | As above | | | |
| Death from hypoglycaemia | As above | | | |
| Fatal myocardial infarction | As above | | | |
| Nonfatal myocardial infarction | As above | | | |
| Angina/ischemic heart disease | 411-414 | I20 or I25 |  |  |
| Heart failure | 427.0 or 427.1 or 427.99 or 428 | I50 |  |  |
| Fatal stroke | As above | | | |
| Nonfatal stroke | As above | | | |
| Nonfatal renal failure | As above | | | |
| Fatal renal disease | As above | | | |
| Amputation | As above | | | |
| Fatal peripheral vascular disease | As above | | | |
| Vitreous haemorrhage | As above | | | |
| Retinal photocoagulation | As above | | | |
| Blindness | 379 | H54.0 or H54.1 or H54.4 |  |  |
| Cataract excision |  |  | 17000 or 171 or 1720 or 1721 or 1723 or 1726 | CJC or CJD or CJE |

^*^ The Danish National Death Registry and the National Hospital Discharge Registry changed coding from ICD-8 to ICD-10 on 1 January 1994. The Danish National Death Registry contains only the first 4 characters of the ICD codes, while the National Hospital Discharge Registry contains all 5 characters.

^†^ The National Hospital Discharge Registry changed coding of surgical procedures from the 3^rd^ edition of The Danish Classification of Surgical Procedures to the Nordic Classification of Surgical Procedures on 1 January 1996.

Figure S1


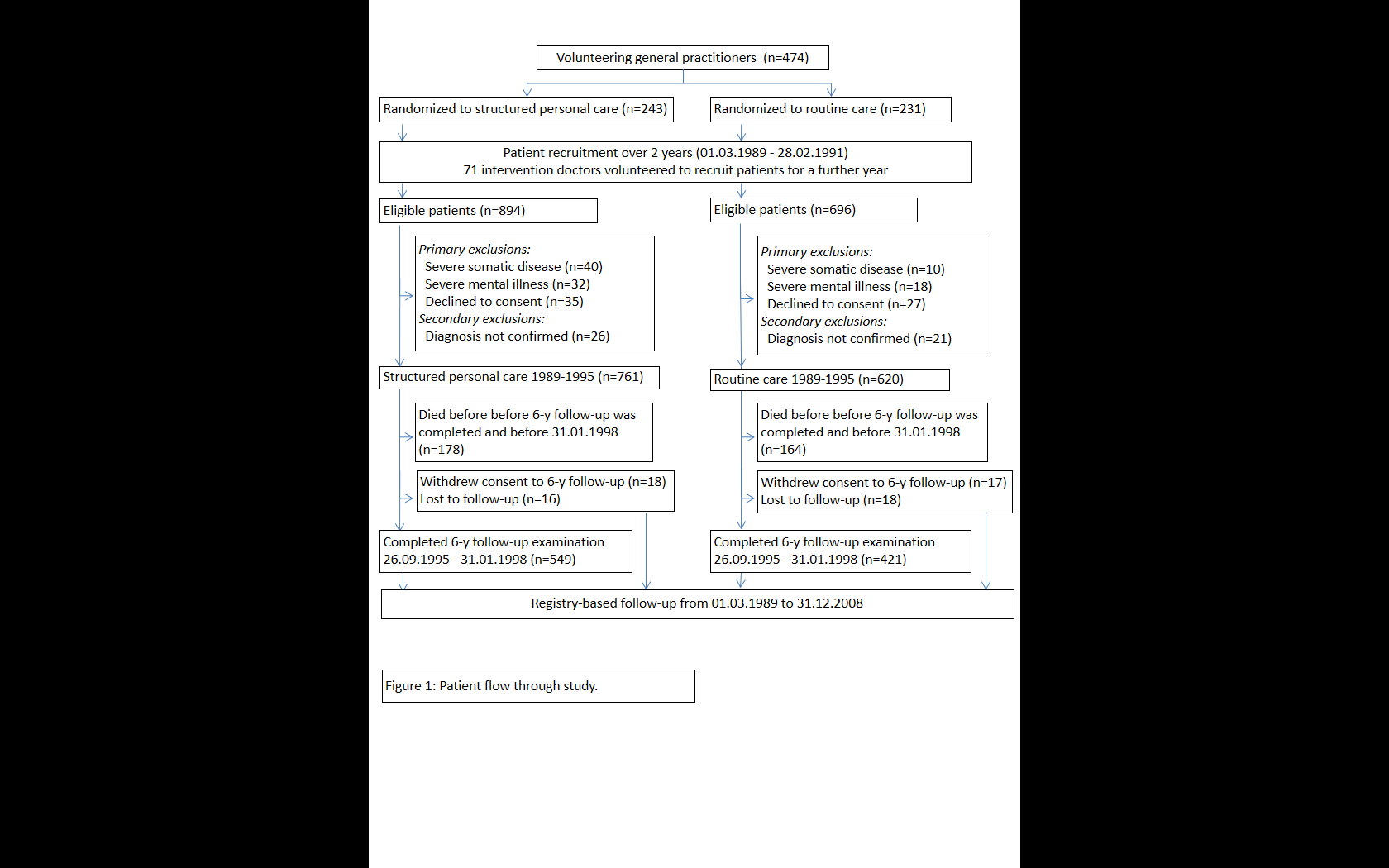

Supplement: Supplementary file 1 — Table S1. Categorization of all patients from the Diabetes Care in General Practice (DCGP) study with ICD-10 diagnoses in the Danish Cancer Register (DCR). Table S2. Definition of clinical outcomes in the 19-year registry-based monitoring of the Diabetes Care in General Practice (DCGP) study. Figure S1. Patient flow through study. (DOCX 110 kb) [file 12902_2019_377_MOESM1_ESM.docx]
